# Calcium pyrophosphate dihydrate deposition disease in the temporomandibular joint: diagnosis and treatment

**DOI:** 10.1186/s40902-018-0158-0

**Published:** 2018-08-03

**Authors:** Kwang-Jun Kwon, Hyun Seok, Jang-Ha Lee, Min-Keun Kim, Seong-Gon Kim, Hyung-Ki Park, Hang-Moon Choi

**Affiliations:** 1 Department of Oral and Maxillofacial Surgery, College of Dentistry, Gangneung-Wonju National University, Gangneung, 25457 Republic of Korea; 2 Department of Oral and Maxillofacial Surgery, Chungbuk National University Hospital, Cheongju, 28644 Republic of Korea; 3 Gangwon Regional Division, Korea Institute of Industrial Technology, Gangneung, 25440 Republic of Korea; 4 Department of Oral and Maxillofacial Radiology, College of Dentistry, Gangneung-Wonju National University, Gangneung, 25457 Republic of Korea

**Keywords:** Calcium pyrophosphate dihydrate deposition disease, Pseudogout, Ramus osteotomy, Scanning electronic microscopy/energy-dispersive X-ray spectroscopy

## Abstract

**Background:**

Calcium pyrophosphate dihydrate deposition disease (CPDD) is a rare disease in the temporomandibular joint (TMJ) space. It forms a calcified crystal mass and induces a limitation of joint movement.

**Case presentation:**

The calcified mass in our case was occupied in the left TMJ area and extended to the infratemporal and middle cranial fossa. For a complete excision of this mass, we performed a vertical ramus osteotomy and resected the mass around the mandibular condyle. The calcified mass in the infratemporal fossa was carefully excised, and the segmented mandible was anatomically repositioned. Scanning electronic microscopy (SEM)/energy-dispersive X-ray spectroscopy (EDS) microanalysis was performed to evaluate the calcified mass. The result of SEM/EDS showed that the crystal mass was completely composed of calcium pyrophosphate dihydrate. This result strongly suggested that the calcified mass was CPDD in the TMJ area.

**Conclusions:**

CPDD in the TMJ is a rare disease and is difficult to differentially diagnose from other neoplasms. A histological examination and quantitative microanalysis are required to confirm the diagnosis. In our patient, CPDD in the TMJ was successfully removed via the extracorporeal approach. SEM/EDS microanalysis was used for the differential diagnosis.

## Background

Calcium pyrophosphate dihydrate deposition disease (CPDD) is a rare arthropathy that includes a calcium pyrophosphate crystal deposition in the articular space [1]. CPDD has been termed as “pseudogout” because CPDD patients show gout-like symptoms and calcium pyrophosphate crystal deposition in the synovial fluid without sodium urate [2]. The etiology of CPDD is unknown. Recently, it has been proposed to result from a metabolic disorder related to phosphate metabolism [3]. The predominant site of occurrence of CPDD is the knee and wrist. Involvement of the temporomandibular joint (TMJ) has been rarely reported [4]. The involvement of CPDD in the TMJ shows various clinical symptoms, such as pre-auricular pain, swelling, and trismus due to the crystal mass [1]. The presence of the calcium pyrophosphate crystal deposit forms a tumor mass in the TMJ and leads to a limitation of condylar movement [5]. The common treatment of CPDD in the TMJ is surgical excision of the mass for the recovery of joint function. The recurrence of this tumor has been rarely reported [6].

We experienced extensively involved CPDD in the left TMJ. The calcified mass of CPDD was extended not only in the left TMJ space but also in the middle cranial and infratemporal fossa area. For a total excision of the mass in the middle cranial fossa, a more extensive surgical approach was required. To completely remove the mass from the infratemporal fossa, we performed a vertical ramus osteotomy and repositioned the segmented condyle after mass excision. In addition, scanning electronic microscopy (SEM)/energy-dispersive X-ray spectroscopy (EDS) microanalysis was performed to analyze the crystal deposit and diagnose the mass.

## Case presentation

**Fig. 1:**
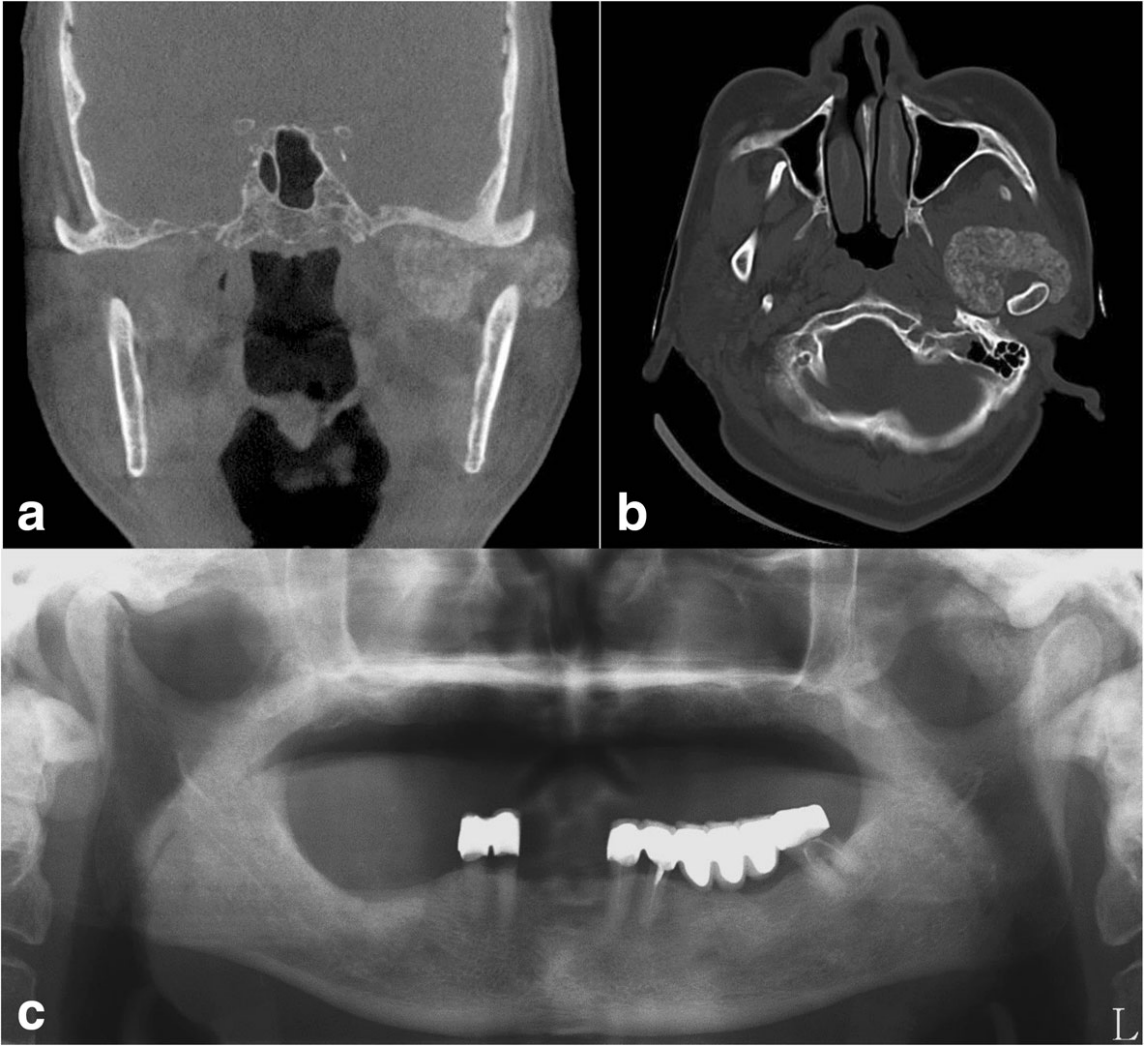
A well-defined calcified mass in the left temporomandibular joint space and infratemporal fossa. **a** Coronal view and **b** axial view of CBCT and **c** preoperative panoramic view

A 72-year-old male visited Gangneung-Wonju Dental Hospital due to pain and induration of the left pre-auricular area. He had no specific medical history and discomfort and crepitus during mouth opening that had persisted for a couple of years. Recently, he suffered from pain upon palpation of the left pre-auricular area. The patient had a mild limitation of mouth opening that was 30 mm. A radiologically, well-defined calcified mass was observed surrounding the left mandibular condyle in cone beam computed tomography (CBCT) images (Fig. 1a). The mass was 49 × 35 × 25 mm in size and encompassed the mandibular condyle as a round shape. It occupied the infratemporal fossa and parotid gland area (Fig. 1b) and medially extended to the area near the pterygoid plate of the sphenoid bone. Due to the extension of the mass, the mandible condyle was laterally displaced. The margin of the mass was clear, and small calcified materials were distributed throughout the mass. An infiltrative sign to the surrounding tissue was not observed. This case report was approved by the institutional review board (IRB) of Gangneung-Wonju National University Dental Hospital (2017-018).

### Surgical excision with vertical ramus osteotomy of the mandible and repositioning of the condyle

**Fig. 2:**
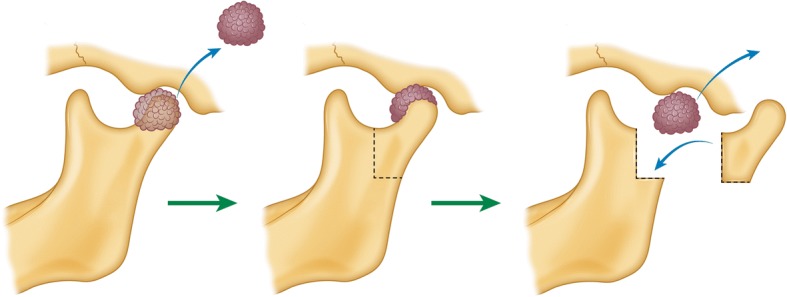
Illustration of the surgical process. The lateral portion of the mass was initially removed. Next, the condylar process was removed by vertical ramus osteotomy of the condyle. The medial portion of the mass was accessed after removing the condylar process. After removal of the medial mass, the condylar segment was anatomically repositioned

This mass was assumed to be a benign calcifying lesion, such as pseudogout or synovial chondromatosis. We decided to surgically excise the mass and perform a biopsy under general anesthesia. We planned a surgical excision of the mass on the lateral aspect of the condyle using a pre-auricular approach. In addition, for complete excision of the mass, the medial aspect of the condyle and infratemporal fossa area was accessed by resecting the condyle after vertical ramus osteotomy of the mandible. After complete excision of the mass, the separated condyle segment was repositioned to its original location and fixed with a titanium plate (Fig. 2).

**Fig. 3:**
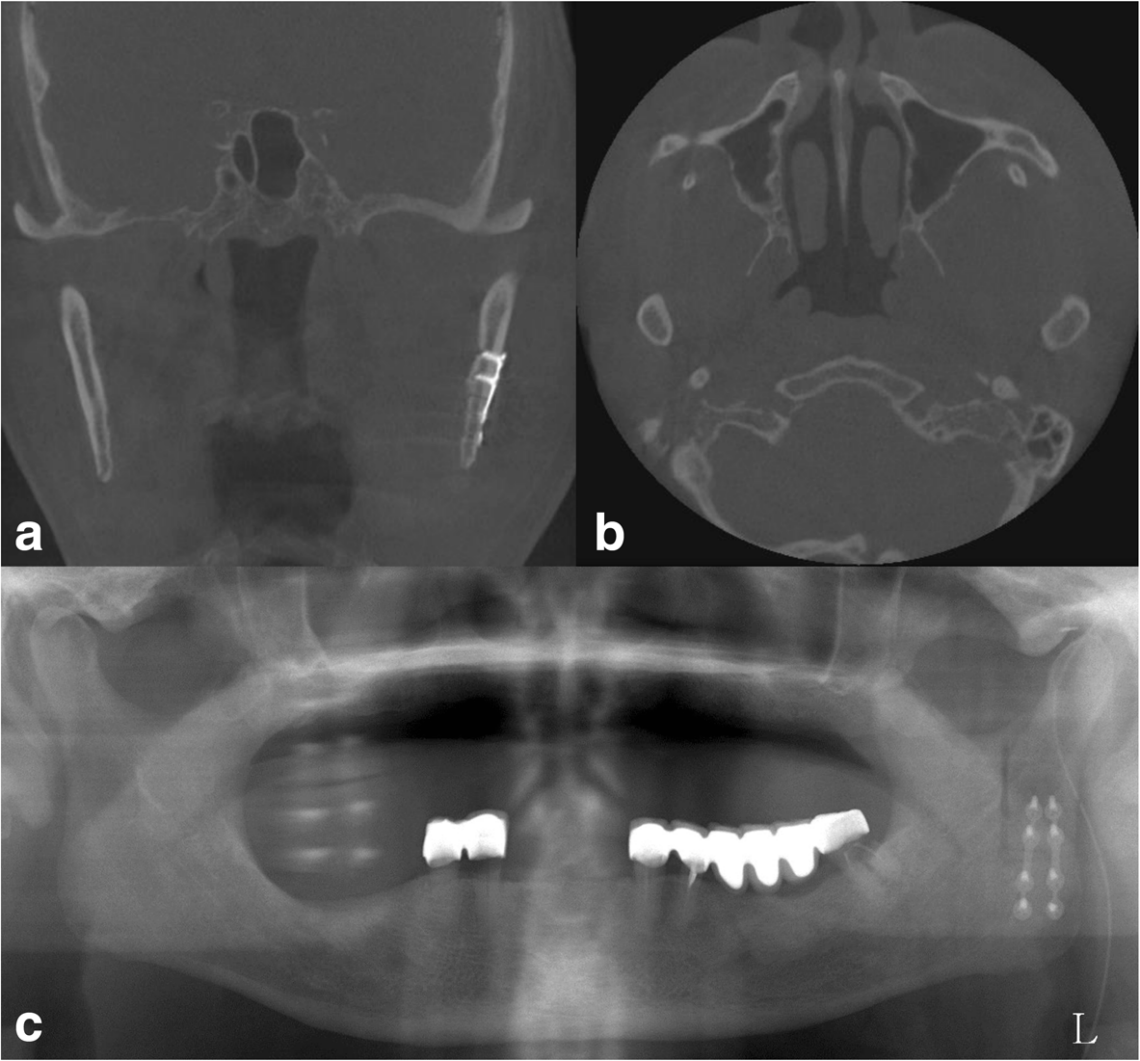
**a** Anatomical repositioning of the segmented condyle after mass excision and **b** complete excision of the mass from the infratemporal fossa in CBCT. **c** Panoramic view after operation

The pre-auricular incision was performed and slightly extended to the left retromandibular area to approach the left mandible angle area, similar to an S shape. The skin flap was raised to expose the superior, anterior, and inferior border of the mass. The mass was bluntly dissected from adjacent soft tissue to prevent facial nerve injury. One half of the mass was first removed by excision, and then, the lateral surface of the condylar process was exposed. Before vertical osteotomy of mandible ramus, two titanium mini-plates were pre-drilled and adapted to the lateral surface of the ramus for accurate repositioning of the condyle segment. In addition, all of the titanium mini-plates and screws were removed. L-shaped ramus osteotomy was performed from the sigmoid notch to the posterior border of the mandible with a reciprocating saw. After extracorporeal removal of the condyle segment, the residual half of the mass on the medial aspect of the condyle and infratemporal fossa was carefully dissected with the pterygoid muscles and excised without damaging the surrounding vessel and nerve tissue. Next, the condyle segments were repositioned and fixed to the remaining mandible body with two titanium plates and mini screws (Fig. 3). The defect was filled with a gelatin sponge, and then, a layered suture was performed. After surgery, the patient showed transient weakness of the facial nerve. Otherwise, the 3-month post-operative follow-up was uneventful and he shows normal jaw movement.

### Histopathological examination

**Fig. 4:**
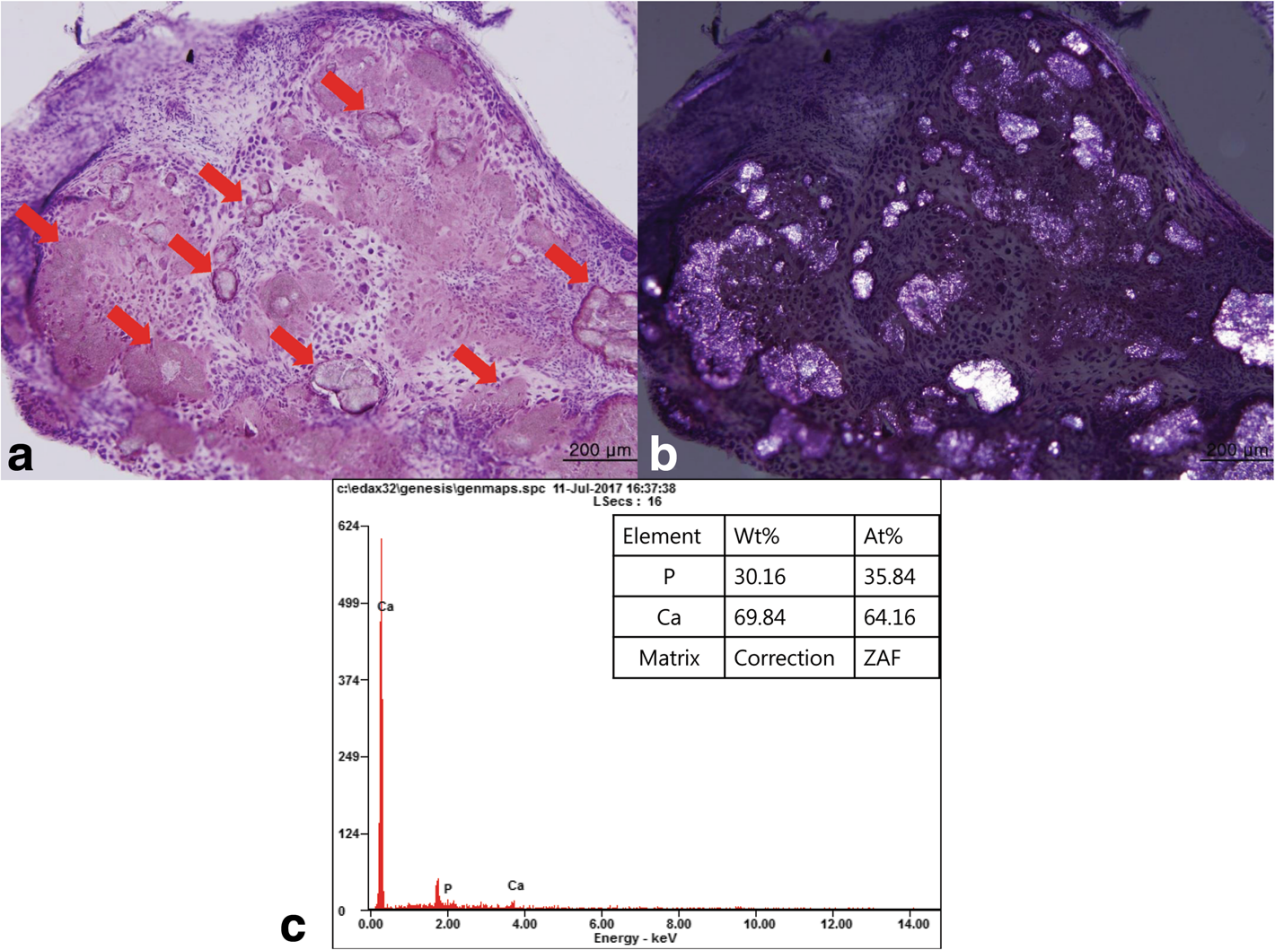
Histological examination and scanning electron microscope (SEM) image/energy-dispersive X-ray spectroscopy (EDS) microanalysis of the calcium pyrophosphate crystal. **a** Rod- and rhomboid-shaped crystal deposits were observed via hematoxylin and eosin staining (red arrow). **b** The crystal deposit is shown to have birefringence under polarized light (original magnification × 100). **c** SEM/EDS spectrum of the CPPD crystal

The excised specimen was stained with hematoxylin and eosin (H&E) for a histopathological examination and was observed using a polarized microscope under polarized light. This amorphous tissue showed abundant basophilic crystal deposit material in the connective tissue stroma. The material of this crystal deposit had a rod and rhomboid shape and was mostly surrounded by histiocyte, fibroblast, mononuclear inflammatory, and multinucleated giant cells (Fig. 4a). Phagocytosis by multi-nucleated giant cells was frequently observed around the crystal material. The weak strained crystal materials formed tophi and exhibited birefringence in polarized light. The rhomboidal shape of the crystal materials was prominently observed in a polarized microscope view (Fig. 4b). The length of the crystal was approximately 10–20 μm, and its diameter was 1–2 μm. The histological features strongly suggested CPDD in the joint space. Furthermore, the histological diagnosis was suggested as CPDD on the left TMJ.

### Scanning electronic microscopy (SEM)/energy-dispersive X-ray spectroscopy (EDS) microanalysis

SEM/EDS microanalysis was performed to analyze the element composition of the crystal deposit in the specimen. The paraffin block-embedded specimen was sectioned into 5-μm sections and de-paraffinized. Unstained sections were coated with 0.7 nm of OsO_4_ (HPC-1SW; Vacuum Device, Inc., Mito city, Japan). The specimen was observed using a scanning electron microscope (Quanta FEG 250, FEI, Oregon, USA), and its chemical composition was analyzed using an energy-dispersive spectrometer (EDS) (Octane Elite EDS, EDAX, New Jersey, USA) attached to the SEM.

The result of the SEM/EDS microanalysis was shown in Fig. 4c. The EDS spectrum showed peaks of calcium (Ca) and phosphorus (P) in the crystal deposit. The first Ca peak has a similar energy position with oxygen. And the high intensity of the first peak would be attributed to the oxygen peak. Oxygen concentration cannot analyze quantitatively by EDS method; thus, it was excluded in the chemical concentration result. The EDS data was adjusted by the ZAF correction, which considers atomic number, self-absorption, and fluorescence effect to eliminate the atomic number effect on the quantitative analysis of chemical composition. The Ca and P weights in the crystal deposit were 69.84 and 30.16%, respectively (Fig. 4c). The EDS spectrum indicated that the crystal deposit consisted of calcium pyrophosphate dihydrate. The EDS mapping signal showed the distribution of Ca and P elements in the surface of the crystal deposit (data not shown). The high proportion of Ca and P in the crystal deposits further supported the histological diagnosis of CPPD in the left TMJ.

### Discussion

CPDD is a rare pathology in the articular tissue and forms a calcified crystal mass in the synovial membrane [7]. CPDD has been found in a local or generalized form [1]. Local CPDD can be observed in degenerative or necrotic tissue, resulting from a secondary trauma [1]. Generalized CPDD is associated with metabolic disorders, such as hyperparathyroidism, hypothyroidism, hypomagnesemia, and hyperphosphatemia [6]. In addition, diabetes mellitus increases the incidence of CPDD [1]. The site that is predominantly involved is a relatively large joint, such as the knee, shoulder, hip, and wrist of the hand, and sites that are less commonly involved are small joints, such as the TMJ [6]. In CPDD of the TMJ, the calcified crystal mass occupies the TMJ space and induces pre-auricular swelling, pain, tenderness, and limitation of the joint movement [8]. In our case, the patient experienced discomfort and crepitus of the left TMJ a couple of years ago. Recently, he suffered from pain in the left pre-auricular area and limitation of mouth opening.

CPDD in the TMJ should be differentiated from other neoplastic disorders [9]. Radiographically, CPDD in the TMJ appears with small, multiple, and radio-opacity nodules around the TMJ [10]. Occasionally, the calcified radio-opaque mass is extended into adjacent areas, such as the skull base or middle cranial fossa [1]. The radiological findings of CPDD in the TMJ are non-specific and are difficult to differentiate from other diseases by clinical and radiographic findings [6, 11]. The radiological feature of CPDD mimics other neoplasms such as synovial chondromatosis, osteochondroma, and chondroblastoma or malignant tumors [6, 12]. The calcified mass observed in our case occupied the left TMJ space and extended into the infratemporal fossa. The mass was well-defined and consisted of small calcified particles (Fig. 1). The mass was a suspicious, benign calcifying lesion that included CPDD or synovial chondromatosis. A biopsy and histological examination were recommended to confirm the diagnosis.

Microscopically, CPDD is characterized by the deposition of a basophilic calcium pyrophosphate dihydrate crystal in the joint space [1]. The crystal in CPDD has a rhomboid structure and is birefringent under polarized light [13]. The birefringence is a key differential diagnostic tool between gout and CPDD. The gout crystal demonstrates negative birefringence [14]. The specimen in our case had numerous rod- and rhomboid-shaped crystals that were surrounded by histiocyte and giant cells (Fig. 4). In addition, the crystals had a strongly positive birefringence under a polarized microscope. Consistent with these findings, it was strongly suggested that CPDD occurred in the TMJ. However, other crystal materials, such as calcium oxalate, synthetic steroids, and ethylenediaminetetraacetic acid (EDTA), can be positive birefringent under polarized light [9]. For a definitive diagnosis of CPDD, other quantitative and chemical analyses were performed [15]. Electron probe microanalysis can be used to analyze the composition of the crystal. In addition, this technique has been used to detect Ca and P in specimens and for differential diagnosis of CPDD [13].

In our case, SEM/EDS microanalysis was used to analyze the elemental composition of the crystal. The SEM/EDS results showed that the crystal mass completely consisted of calcium pyrophosphate dihydrate. The weight composition of the crystal was 69.84% of Ca and 30.16% of P (Fig. 4c). In a previous study, CPDD occurring in the TMJ showed numerous crystalline deposits and consisted of 100% calcium pyrophosphate dihydrate in infrared spectrophotometry [16]. In addition, the CPDD crystal in the left TMJ showed a high concentration ratio of Ca and P in the SEM/EDS microanalysis [6]. The composition of Ca and P in our specimen confirmed the diagnosis of CPDD. Diagnosis of CPDD should be based on quantitative and chemical analyses. Using SEM/EDS microanalysis, we analyzed the composition of the crystal mass and performed a differential diagnosis of CPDD with other diseases.

The infratemporal fossa is a highly complex anatomical region that has various nerves and vessels exiting from the foramina of the skull base [17]. The inferior boundary of infratemporal fossa is the medial pterygoid muscle and the temporal bone locates in the superior and posterior region of the infratemporal fossa. Superiorly, it is bounded by a greater wing of sphenoid bone and, anteriorly, by the posterior border of the maxillary sinus [17]. The medial border consists of the pterygopalatine fossa and pterygoid plate [18]. Laterally, the zygomatic arch, temporalis muscle, condyle, parotid gland, and facial nerve are surrounded [19]. The surgical approach to the infratemporal fossa has been performed by the pre-auricular and temporal approach [19]. Osteotomy of the zygomatic arch, mandible condyle, and coronoid process has been performed for surgical access to the middle cranial fossa [17, 20]. The mandibular condylar process is temporarily resected to the access of the infratemporal fossa and excised the chondrosarcoma [21]. After osteotomy of the mandible condyle, the internal maxillary artery, pterygoid muscle, and mandibular nerve can be accessed [17].

The crystal deposit mass in our case extended to the infratemporal fossa and lateral pterygoid plate area in the CBCT (Fig. 1). Condylar resection was required to access the infratemporal fossa and for complete excision of the mass. Before vertical osteotomy of the mandibular condylar process, plates and screws were pre-drilled on the condylar neck and mandible ramus area. Next, osteotomy was performed from the sigmoid notch to the posterior border of the mandible. After resection of the condyle segment, the crystal mass was carefully dissected from the surrounding structure including the internal maxillary artery, pterygoid muscles, and mandibular nerve (Fig. 2). After excision of the mass, the segmented condyle was anatomically repositioned with the pre-drilled plate and screws (Fig. 3). After the operation, the condyle segment was successfully repositioned and the patient showed normal jaw movement and mouth opening. The patient showed transient weakness of the facial nerve. Otherwise, the post-operative follow-up was uneventful.

## Conclusions

In conclusion, the CPDD in the TMJ is a rare disease and is difficult to differentially diagnose from other neoplasms. A histological examination and quantitative microanalysis are required to confirm the diagnosis. In our patient, CPDD in the TMJ was successfully removed using the extracorporeal approach. SEM/EDS microanalysis was helpful for the differential diagnosis.

## Abbreviations

CBCT: Cone beam computed tomography
CPDD: Calcium pyrophosphate dihydrate deposition disease
EDTA: Ethylenediaminetetraacetic acid
H&E: Hematoxylin and eosin
SEM/EDS: Scanning electronic microscopy/energy-dispersive X-ray spectroscopy
TMJ: Temporomandibular joint
